# Proxy indicators to estimate appropriateness of antibiotic prescriptions by general practitioners: a proof-of-concept cross-sectional study based on reimbursement data, north-eastern France 2017

**DOI:** 10.2807/1560-7917.ES.2020.25.27.1900468

**Published:** 2020-07-09

**Authors:** Nathalie Thilly, Ouarda Pereira, Jeroen Schouten, Marlies EJL Hulscher, Céline Pulcini

**Affiliations:** 1 Université de Lorraine, Adaptation, mesure et évaluation en santé. Approches interdisciplinaires (APEMAC), Nancy, France; 2 Université de Lorraine, Centre Hospitalier Régional Universitaire de Nancy (CHRU-Nancy), Département Méthodologie, Promotion, Investigation, Nancy, France; 3 Direction Régionale du Service Médical du Nord-Est, Nancy, France; 4 Radboud University Medical Center, Radboud Institute for Health Sciences, Scientific Center for Quality of Healthcare (IQ healthcare), Nijmegen, the Netherlands; 5 Université de Lorraine, Centre Hospitalier Régional Universitaire de Nancy (CHRU-Nancy), Département de maladies infectieuses, Nancy, France

**Keywords:** antibiotic stewardship, benchmarking, performance, primary care, general practitioners, prescriptions

## Abstract

**Background:**

In most countries, including France, data on clinical indications for outpatient antibiotic prescriptions are not available, making it impossible to assess appropriateness of antibiotic use at prescription level.

**Aim:**

Our objectives were to: (i) propose proxy indicators (PIs) to estimate appropriateness of antibiotic use at general practitioner (GP) level based on routine reimbursement data; and (ii) assess PIs’ performance scores and their clinimetric properties using a large regional reimbursement database.

**Methods:**

A recent systematic literature review on quality indicators was the starting point for defining a set of PIs, taking French national guidelines into account. We performed a cross-sectional study analysing National Health Insurance data (available at prescriber and patient levels) on antibiotics prescribed by GPs in 2017 for individuals living in north-eastern France. We measured performance scores of the PIs and their case-mix stability, and tested their measurability, applicability, and room for improvement (clinimetric properties).

**Results:**

The 3,087 GPs included in this study prescribed a total of 2,077,249 antibiotic treatments. We defined 10 PIs with specific numerators, denominators and targets. Performance was low for almost all indicators ranging from 9% to 75%, with values < 30% for eight of 10 indicators. For all PIs, we found large variation between GPs and patient populations (case-mix stability). Regarding clinimetric properties, all PIs were measurable, applicable, and showed high improvement potential.

**Conclusions:**

The set of 10 PIs showed satisfactory clinimetric properties and might be used to estimate appropriateness of antibiotic prescribing in primary care, in an automated way within antibiotic stewardship programmes.

## Introduction

The vast majority of antibiotics for human use are prescribed in primary care, in particular by general practitioners (GPs), who prescribe ca 70% of total antibiotics used in France in the outpatient setting. The most common indications are respiratory tract infections (around two thirds of overall antibiotic use), followed by urinary tract infections and skin/soft tissue infections [1]. To ensure that antibiotics are used appropriately in various settings, antibiotic stewardship programmes have been introduced [2].

Metrics are important in antibiotic stewardship programmes as they allow to set targets for improvement and give professionals and other stakeholders insight into the current antibiotic use. Many metrics on antibiotic use are available, including both quantity and quality metrics [3-6]. A quantity metric is usually defined as a measure that reflects the volume of antibiotic use. Quantity metrics can often be easily calculated, but it is difficult to identify improvement targets from these data as they only give a rough indication about whether there is a problem with use. Quality metrics or quality indicators (QIs), on the other hand, reflect the degree to which an antibiotic prescription is correct or appropriate and they provide concrete targets for improvement. When using QIs, one needs to define numerators and denominators. The denominator usually describes the target population in absolute numbers, e.g. the number of patients who should receive the recommended care and the numerator represents the population, in absolute numbers, who actually receives recommended care. Quality indicators require information on the clinical indication/diagnosis serving as basis for calculations, which is not the case for quantity metrics [3-6]. A survey conducted in 20 European countries in 2017, showed that computerised national systems routinely linking antibiotic prescriptions to clinical diagnoses were available in only two countries (Turkey and Croatia) [7].

Some metrics can, however, be in a ‘grey zone’, sharing characteristics of both quantity metrics and QIs. These ‘proxy’ indicators (PIs) may be derived from quantity metrics without using clinical indication data. They may still indirectly reflect appropriateness of antibiotic use, provided that they are associated with a clear target. This is for example the case for seasonal variation of antibiotic use, since overprescription in the winter might possibly be the result of unnecessary antibiotic use for viral infections [3,5]. Quality indicators accurately reflect the appropriateness of each antibiotic prescription, whereas PIs can only strongly suggest that antibiotic use at an aggregated level (not the prescription) is appropriate or not, depending on whether the set target is met or not.

A literature review and structured consensus procedure conducted in 2015–16, among international stakeholders from four different groups i.e. (i) medical community; (ii) public health and patients; (iii) antibiotic Research and Development; (iv) payers, policymakers, government and regulators, identified QIs that could be useful at global level to assess the appropriateness of antibiotic prescriptions in the outpatient setting [3]. Given the large number of prescribers involved, and since manual extraction of data is very time-consuming, automated monitoring of QIs is probably the ideal approach. In France, routine outpatient antibiotic reimbursement data are easily available in national and regional databases, providing data on the quantity of antibiotics prescribed by GPs and dispensed by community pharmacies. As in most countries, data on clinical indications for these outpatient antibiotic prescriptions are not available. To explore whether such routine data on the quantity of antibiotics can be used to estimate the appropriateness of antibiotic prescription by individual GPs, and taking the recent overview of outpatient quality indicators as a starting point [3], our objectives were to: (i) define PIs for appropriateness of antibiotic use based on these routine data; and (ii) assess the PIs’ performance scores and their clinimetric properties using a large regional reimbursement database. These PIs should strongly suggest whether antibiotic prescription, in this proof-of-concept study, is appropriate or not in France at GP level.

## Methods

### Study setting and population

We performed a cross-sectional study analysing data regarding antibiotics prescribed by GPs in 2017 for individuals living in Lorraine and Champagne-Ardenne (two regions of north-eastern France), with a population of 2,346,000 and 1,339,270 inhabitants, respectively, according to the 2014 census (total of 66.3 million inhabitants in France) [8]. We included GPs practising in these two regions in 2017, who took care of at least 100 individual patients and wrote at least 10 prescriptions of antibiotics during the year. We then excluded GPs who practised exclusively alternative medicine, such as homoeopathy or acupuncture.

### Data source and study design

In France, individuals pay health service fees, which are refunded by the national health insurance (NHI). Every inhabitant, irrespective of their income and professional status, nationality or age, is covered by the NHI programme.

Data regarding the quantity of antibiotics dispensed by community pharmacies are easily available in the NHI databases, as all antibiotics are reimbursed by the French NHI. Each time a prescribed antibiotic is dispensed to a given patient, information on the medication dispensed, the prescriber (professional identification number) and the patient (identification number) are recorded and electronically sent to the patient’s Regional Health Insurance Fund. Using the prescriber and the patient identification numbers, further information is then available in the NHI databases, such as the prescriber’s specialty or the patient’s main individual characteristics (age, sex, presence of chronic disease or low income, place of residence). However, as previously mentioned, the NHI databases do not provide any information about clinical indications/diagnoses related to the specific prescription.

Data included each occasion on which systemic antibiotics (J01 code according to the Anatomical Therapeutic Chemical (ATC) 2017 classification system [9]) were prescribed by eligible GPs and dispensed by community pharmacies during the year. They were collected from the outpatient reimbursement database of the north-eastern France Regional Health Insurance Fund (Grand Est – CNAM) as part of its routine work. This fund covers salaried workers and their families but also other socio-professional groups (such as those unemployed) and accounted for 94.5% of the population in 2017.

### Selection and operationalisation of potential proxy indicators

A comprehensive overview of 32 existing quality indicators (QIs) for appropriate use of antibiotics in the outpatient setting was published in 2018 [3]. Based on this exhaustive review, the authors, with input from GP colleagues, adapted those indicators that could be operationalised (i.e. translated into numerators/denominators, with targets) using routine reimbursement data, and that could serve as proxy to measure the appropriateness of antibiotic prescriptions in France at GP level. The scientific evidence base (using national guidelines whenever possible) supporting the definition of each proxy indicator is detailed in the supplementary Table S1.

### Appropriateness of general practitioners’ antibiotic prescription and its variability using proxy indicators

Each PI was calculated at GP level to assess current appropriateness of antibiotic prescribing by French GPs and its variability. We developed fractions (numerator/denominator) of certain prescriptions and used thresholds/cut-off values to indicate high quality of care. For each PI, we defined a target to be reached when practices are *optimal* i.e. 100% compliant with French guidelines and (when relevant) a less restrictive target reflecting *acceptable* practices. The acceptable targets were introduced since guidelines sometimes do not apply to specific patients for example patients with multidrug-resistant bacteria, or with contra-indications to first-line treatments [10-12]. We created an aggregated GP score for each PI, based on aggregated prescriptions made by a GP for their patients. The unit of measurement at patient level was the antibiotic treatment, i.e. the antibiotic dispensed on a given day in 2017 by a community pharmacy following a prescription by a GP. If two different antibiotics were prescribed by a GP and dispensed on the same day, they were counted separately.

With the objective to study the variability of PIs, subgroup analyses were performed to determine whether PI scores were similar across different patient populations or not (case-mix stability). These analyses show whether it is necessary, for example when comparing an individual GP’s scores over time or when comparing various GPs, to look at various specific patient subgroups, or not. The effects on the PI scores of the following patient factors were studied: age (> 65 years old), presence of a chronic disease (‘*affection de longue durée*’, identified by the NHI because of exemption from health insurance co-payments), presence of low income (identified using coverage by the public supplementary health insurance programme), and living in a nursing home.

**Clinimetric properties of the potential proxy indicators**

The following clinimetric properties were evaluated in order to demonstrate their value as measurement instruments to assess the appropriateness of antibiotic prescription in routine practice [37,38]:

- *Measurability*: Measurability was defined as the availability of data required to calculate the indicator. An indicator was considered measurable if data necessary to calculate the PI score were available for more than 75% of prescriptions/patients, i.e. data were missing in < 25% of cases.
- *Applicability*: The PI was considered applicable if the score was meaningful for the GP, i.e. it reflected at least 10 clinical situations. In practice, a PI score could not be calculated for a given GP if (i) less than 10 prescriptions/patients were identified for the denominator for PIs focusing on suboptimal practices i.e. drugs that should not be prescribed (e.g. PI 1, 3, 7, 8, 9, 10), where the optimal target value was close to 0, and (ii) less than 10 prescriptions were identified for either the numerator or the denominator for PIs describing both suboptimal and good practices (e.g. PI 2, 4, 5, 6), with the denominator being different from null. Overall, a PI was considered applicable if it could be calculated from data extracted for more than 75% of the GPs.
- *Potential room for improvement*: Potential room for improvement measured the sensitivity of a PI to detect variability in appropriateness of prescriptions between physicians and over time. It was expressed as 100% minus the performance score, with performance expressing the percentage of GPs who reached the PI target. High performance scores make indicators less sensitive and therefore less useful in routine practice, so the potential room for improvement for a PI was considered as low if it was ≤ 15%. In our study, we considered the acceptable target of each PI to estimate the improvement potential.

GP: general practitioner; PI: proxy indicator.

### Statistical analysis

PIs’ results were first calculated for each individual GP, and then we calculated medians and interquartile ranges (IQR) for all eligible GPs. The performance scores, i.e. the percentage of GPs who reached the optimal and the acceptable targets, were also estimated. Measurability, applicability and improvement potential are presented as percentages and case-mix stability as potential room for improvement for the specific patient populations. All analyses were performed with SAS Enterprise Guide version 7.1 (SAS Institute Inc., Cary, North Carolina, United States).

### Ethical statement

As our study was retrospective and did not modify the medical care of patients, and complete anonymity was preserved at both patient and physician levels, no ethical committee approval was required, in accordance with the French law.

## Results

### Characteristics of general practitioners

Of the 3,158 GPs practising in the Lorraine and the Champagne-Ardenne regions, we included 3,087 GPs who met the inclusion criteria. Their mean age was 53.2 years (standard deviation (SD): 10.7) and 67% were men. They took care, on average, of 1,388 (SD: 653) different patients in 2017 (a patient can, in France, visit several GPs a year), with a total of 1,696 consultations (SD: 869). The 3,087 GPs included prescribed in 2017 a total of 2,077,249 antibiotic treatments that were dispensed to 1,335,401 individual patients. Regarding their overall patients’ characteristics, 51% (1,690,779/3,328,307) were male, 20% (655,010/3,328,307) were aged < 16 years, 18% (612,408/3,328,307) were aged > 65 years, 0.8% (26,626/3,328,307) lived in a nursing home, 14% (477,878/3,328,307) had a chronic disease and 11% (369,109/3,328,307) were in the low-income category.

### Selection and operationalisation of potential proxy indicators

We operationalised 10 PIs, which are described in Table 1 with their numerators, denominators and targets (further details are available in the supplementary Tables S1 and S2) [13]. Six PIs (PI 2, PI 6, PI 7, PI 8, PI 9, PI 10) were original i.e. not reported in the literature before, to the best of our knowledge (supplementary Table S3 and [3-7]), two were based on existing numerators and denominators, but we added the definition of the target (PI 1, PI 3), and two were already published indicators (PI 4, PI 5).

**Table 1:** List of proxy indicators to estimate the appropriateness of systemic antibiotic prescriptions by general practitioners, north-eastern France, 2017

Proxy indicator	Numerator description	Denominator description	Target value	Target patients
**PI 1** Antibiotic prescriptions against UTI in men (ratio)	Number of prescriptions of: nitrofurantoin (J01XE01) + certain (fluoro)quinolones^a^ (J01MB + J01MA06 + J01MA04 + J01MA07) + fosfomycin-trometamol (J01XX01)	100 active^b^ male patients ≥ 16 years old	Optimal target: 0 Acceptable target: < 0.5	Men ≥ 16 years old
**PI 2** Antibiotic prescriptions against UTI in women (ratio)	Number of prescriptions of: nitrofurantoin (J01XE01) + pivmecillinam (J01CA08) + fosfomycin-trometamol (J01XX01)	Number of prescriptions of quinolones (J01M)	Target: > 1	Women ≥ 16 years old
**PI 3** Repeated prescription of quinolones (%)	Number of prescriptions of quinolones (J01M) among patients having been prescribed a quinolone (J01M) in the preceding 6 months	Total number of prescriptions of quinolones (J01M)	Optimal target: 0 Acceptable target: < 10%	Men and women ≥ 16 years old
**PI 4** Seasonal variation of total antibiotic prescriptions (%)	(Number of prescriptions of antibiotics (J01) during the cold-weather season (January–March and October–December) / Number of prescriptions of antibiotics (J01) during the hot-weather season (April–September) – 1) x 100		Target: < 20%	All patients
**PI 5** Seasonal variation of quinolone prescriptions (%)	(Number of prescriptions of quinolones (J01M) during the cold-weather season (January–March and October–December) / Number of prescriptions of quinolones (J01M) during the hot-weather season (April–September) – 1) x 100		Optimal target: < 5% Acceptable target: < 10%	All patients
**PI 6** Amoxicillin / second-line antibiotics prescriptions (ratio)	Number of prescriptions of amoxicillin (J01CA04)	Number of prescriptions of: amoxicillin-clavulanic acid (J01CR02) + quinolones (J01M) + cephalosporins (J01D) + MLSK^c^ (J01F)	Target: > 1	All patients
**PI 7** Prescriptions of not indicated antibiotics (%)	Number of prescriptions of: lomefloxacin (J01MA07), moxifloxacin (J01MA14), certain (fluoro)quinolones^a^ (J01MB + J01MA06 + J01MA04 + J01MA07), telithromycin (J01FA15), spiramycin-metronidazole (J01RA04) and cefaclor (J01DC04)	Total number of antibiotic prescriptions	Optimal target: 0 Acceptable target: < 0.5%	All patients
**PI 8** Estimated duration of antibiotic prescriptions > 8 days (%)	Number of prescriptions > 8 days for the following antibiotics: amoxicillin (J01CA04), co-amoxiclav (J01CR02), cefuroxime, cefpodoxime, roxithromycin, clarithromycin, pristinamycin and nitrofurantoin (J01FG0)	Total number of antibiotic prescriptions for these eight antibiotics (calculation of this metric is explained in detail in supplementary Table S2)	Optimal target: < 5% Acceptable target: < 10%	All patients
**PI 9** Co-prescription of antibiotic and systemic non-steroidal anti-inflammatory drugs (%)	Number of antibiotic(s) (J01) + systemic NSAID(s) (M01A) co-prescribed on the same day	Total number of antibiotic prescriptions	Optimal target: 0 Acceptable target: < 5%	All patients
**PI 10** Co-prescription of antibiotic and systemic corticosteroids (%)	Number of antibiotic(s) (J01) + systemic corticosteroid(s) (H02AB) co-prescribed on the same day	Total number of antibiotic prescriptions	Optimal target: 0 Acceptable target: < 5%	All patients

UTI: urinary tract infections.

^a^ J01MB (rosoxacin, nalidixic acid, piromidic acid, pipemidic acid, oxolinic acid, cinoxacin, flumequine, nemonoxacin), J01MA06 (norfloxacin) + J01MA04 (enoxacin) + J01MA07 (lomefloxacin).

^b^ An active patient is a patient seen at least once by the general practitioner during the year 2017.

^c^ MLSK: macrolides, lincosamides, streptogramins and ketolides.

PI 1 and PI 7 refer to antibiotics that should not be used in routine practice according to national guidelines, i.e. antibiotics that are exclusively used for urinary tract infections but are not indicated in male adults (PI 1), and antibiotics that are not/rarely indicated in any patient as they are not recommended by national guidelines to be used in routine practice (PI 7).

PI 2 and PI 6 focus on preferred prescribing of first-line antibiotics, such as nitrofurantoin, pivmecillinam, and fosfomycin-trometamol rather than quinolones in women, and amoxicillin rather than second-line antibiotics for all patients.

PI 3 refers to the repeated prescription of quinolones within a 6-month period which is discouraged by national guidelines, and PI 8 to the estimated treatment duration which should not exceed 8 days in the vast majority of primary care bacterial infections (see supplementary Table S2 for further details).

As most (upper and lower) respiratory tract infections occurring during the cold-weather season are viral infections, prescription of antibiotics in general (PI 4) and quinolones in particular (PI 5) should be relatively stable during the year and this stability or lack of seasonal variation is assessed by PI 4 and PI 5 [14-16].

The two last PIs (PI 9 and PI 10) are not exclusively focused on antibiotics but on the co-prescription of anti-inflammatory drugs (non-steroidal and steroidal) and antibiotics, that is generally not recommended, since anti-inflammatory drugs are not indicated in bacterial infections according to national guidelines [17]. These two PIs therefore estimate more broadly inappropriate treatment of infections rather than only inappropriate prescription of antibiotics.

For all PIs, a low value indicates high quality of care, except for PI 2 and PI 6 where a high value indicates high quality of care.

### Appropriateness and variability of antibiotic prescriptions

Results concerning the 10 PIs (Table 2) showed that antibiotic prescription practices were far from being optimal and wide variations of prescribing (large IQRs) were observed between GPs. Performance was low for almost all indicators ranging from 9% to 75%, with values < 30% for eight of 10 indicators, with particularly large room for improvement identified for the following six PIs: repeated prescription of quinolones (PI 3), seasonal variation of total antibiotic prescriptions (PI 4), prescriptions of not indicated antibiotics (PI 7), estimated duration of antibiotic prescriptions > 8 days (PI 8), and co-prescription of non-steroidal anti-inflammatory drugs (PI 9) and systemic corticosteroids (PI 10).

**Table 2:** Results for 10 proxy indicators to estimate the appropriateness of systemic antibiotic prescriptions by general practitioners, north-eastern France, 2017

Proxy indicator	Median	IQR	Target value	% of GPs who reached the target (performance)
**PI 1** Antibiotic prescriptions against UTI in men (ratio)	0.2	0; 0.5	Optimal: 0 Acceptable: < 0.5	Optimal: 46.6% Acceptable: 73.1%
**PI 2** Antibiotic prescriptions against UTI in women (ratio)	2.1	1.0; 4.0	> 1	75.0%
**PI 3** Repeated prescription of quinolones (%)	16.7	8.7; 24.1	Optimal: 0 Acceptable: < 10%	Optimal: 13.6% Acceptable: 27.6%
**PI 4** Seasonal variation of total antibiotic prescriptions (%)	52.4	35.9; 69.6	< 20%	10.6%
**PI 5** Seasonal variation of quinolone prescriptions (%)	25.0	−6.7; 71.4	Optimal: < 5% Acceptable: < 10%	Optimal: 34.8% Acceptable: 38.2%
**PI 6** Amoxicillin / second-line antibiotics prescriptions (ratio)	0.8	0.5; 1.2	> 1	35.5%
**PI 7** Prescription of not indicated antibiotics (%)	2.3	0.9; 4.3	Optimal target: 0 Acceptable target: < 0.5%	Optimal: 7.2% Acceptable: 14.6%
**PI 8** Estimated duration of antibiotic prescriptions > 8 days (%)	27.1	16.7; 38.9	Optimal: < 5% Acceptable: < 10%	Optimal: 2.4% Acceptable: 9.1%
**PI 9** Co-prescription of antibiotic and systemic non-steroidal anti-inflammatory drugs (%)	10.8	5.8; 18.6	Optimal: 0 Acceptable: < 5%	Optimal: 1.4% Acceptable: 20.9%
**PI 10** Co-prescription of antibiotic and systemic corticosteroids (%)	13.4	7.6; 21.8	Optimal: 0 Acceptable target: < 5%	Optimal: 1.7% Acceptable: 13.4%

GP: general practitioner; IQR: interquartile range; SD: standard deviation; UTI: urinary tract infection.

The distribution of the 10 PI values among our sample of GPs is presented in supplementary Figure S1.

The PI performances varied according to the patient population for all PIs, even if the PIs and their targets were theoretically clinically relevant in all the situations explored in this case-mix analysis (Table 3).

**Table 3:** Clinimetric properties of 10 proxy indicators to estimate the appropriateness of systemic antibiotic prescriptions by general practitioners and case-mix stability, north-eastern France, 2017

Proxy indicator	Measurability	Applicability		Improvement potential	Case-mix stability
Score or definition	Missing data (%)	n	%	% (100 – (acceptable) performance)	Improvement potential for specific populations
**PI 1** Antibiotic prescriptions against UTI in men (ratio)	0	3,085	99.9%	27.0%	Age > 65: 37.0% Chronic disease: 32.9% Low income: 5.9% Nursing home: 5.7%
**PI 2** Antibiotic prescriptions against UTI in women (ratio)	0	2,873	93.1%	25.0%	Age > 65: 35.3% Chronic disease: 42.2% Low income: 51.1% Nursing home: 73.5%
**PI 3** Repeated prescription of quinolones (%)	0	2,646	85.7%	72.4%	Age > 65: 69.5% Chronic disease: 65.1% Low income: 30.1% Nursing home: 31.5%
**PI 4** Seasonal variation of total antibiotic prescriptions (%)	0	3,054	98.9%	89.3%	Age > 65: 81.4% Chronic disease: 79.9% Low income: 71.4% Nursing home: 51.7%
**PI 5** Seasonal variation of quinolone prescriptions (%)	0	2,404	77.9%	61.8%	Age > 65: 54.1% Chronic disease: 51.3% Low income: 31.2% Nursing home: 20.7%
**PI 6** Amoxicillin / second-line antibiotics prescriptions (ratio)	0	3,051	98.8%	64.4%	Age > 65: 84.5% Chronic disease: 81.8% Low income: 50.4% Nursing home: 87.1%
**PI 7** Prescriptions of not indicated antibiotics (%)	0	3,087	100%	85.4%	Age > 65: 73.8% Chronic disease: 68.9% Low income: 51.6% Nursing home: 12.6%
**PI 8** Estimated duration of antibiotic prescriptions > 8 days (%)	0	3,062	99.2%	90.9%	Age > 65: 95.1% Chronic disease: 94.5% Low income: 71.6% Nursing home: 72.7%
**PI 9** Co-prescription of antibiotic and systemic non-steroidal anti-inflammatory drugs (%)	0	3,087	100%	79.1%	Age > 65: 37.0% Chronic disease: 46.0% Low income: 74.7% Nursing home: 4.3%
**PI 10** Co-prescription of antibiotic and systemic corticosteroids (%)	0	3,087	100%	86.6%	Age > 65: 75.9% Chronic disease: 77.7% Low income: 75.3% Nursing home: 28.6%

UTI: urinary tract infections.

### Clinimetric properties of the proxy indicators

The results for the evaluation of the clinimetric properties measurability, applicability and potential room for improvement are shown in Table 3.

As data required to calculate the PIs were collected from the outpatient reimbursement database of the Regional Health Insurance Fund and as all antibiotics are reimbursed by the NHI, we had no missing data and all the PIs were measurable in 100% of the cases. Some PIs could occasionally not be calculated for some GPs, when the denominator (or the numerator for PIs 2, 4, 5 and 6) was < 10 prescriptions/patients. This mainly concerned the three PIs (PI 2, PI 3 and PI 5) related to prescription of quinolones as some GPs were low prescribers for this class of antibiotics. However, the applicability was always higher than 75%. Overall, PIs’ performances were low, and as a consequence potential room for improvement was considerable, varying from 25.0% (PI 2) to 90.9% (PI 8). Improvement potential was > 50% for eight of the 10 PIs.

## Discussion

Based on reimbursement data only, we measured 10 PIs that estimate the appropriateness of antibiotic prescriptions by French GPs, with good results for the clinimetric properties measurability, applicability and potential room for improvement. Performance was low for almost all indicators, suggesting a large room for improvement. Performance variability was high between GPs, indicated by high SD values, and between different patient groups, indicated by results of the case-mix stability analyses.

Performance levels in our study are comparable to the few studies we identified that used identical or comparable indicators (see supplementary Table S3 for further details) [18-23]. Our set of PIs is broad and includes all antibiotic classes used in general practice, and it thus covers a wide range of potential clinical indications. It encompasses both unnecessary antibiotic prescriptions and inappropriate prescribing, including inappropriate choice of antibiotic when prescribing is warranted, and inappropriate duration of antibiotic treatment. It also includes two ‘positive’ PIs (PI 2 and PI 6) promoting the prescription of first-line treatments.

The developed PIs are easily measurable at national level in France, using the NHI database. They could be used in different ways to reach public health objectives, in the future. With the aim that GPs themselves will use the data to initiate improvement interventions, the PIs could be used to perform automated audits and feedback in almost real time. They could be included into the existing antibiotic profiles with benchmarking at regional level that are sent regularly by the NHI to GPs, comparing their personal data to regional ones. NHI delegates visit all GPs several times a year, to discuss these prescribing profiles and present existing guidelines. However, only crude quantity metrics without targets have been used as feedback so far, for example, total antibiotic prescription plus antibiotic prescription of different antibiotic classes, such as amoxicillin, co-amoxiclav, quinolones, macrolides and cephalosporins. A randomised clinical trial conducted in Switzerland, assessing the impact of quarterly antibiotic prescription feedback using quantity metrics without targets, showed that this intervention was not associated with a change in antibiotic use [24]. It is possible that pure quantity metrics, that do not describe the appropriateness of antibiotic prescriptions, are not relevant enough to GPs. Integrating feedback on PIs into these regular visits, with both explicit targets and a personalised action plan, deserves further investigation [25,26].

The PIs could also be used by regional antibiotic stewardship networks/teams and regional/national health authorities as a screening/diagnostic tool to guide and adapt stewardship interventions (e.g. in-depth audits based on medical records, education, peer group discussion of clinical cases) in their region. In both cases, reviewing performance by specific patient populations can help GPs and antibiotic stewards tailor their actions.

The third option for using the PIs could be to integrate them into the existing NHI pay-for-performance system. Pay-for-performance is a payment model that offers financial incentives to physicians for meeting certain performance measures. If the PIs were to be used for pay-for-performance purposes, it would be necessary to select a specific patient population for each PI, as the case-mix influenced PI scores. There are currently four indicators in place in France, focusing on antibiotic prescribing, and this French programme has led to an almost 4% reduction in antibiotic prescribing for these particular indicators [27]. The English pay-for-performance ‘quality premium’ programme has achieved an 8% reduction in antibiotic prescribing, without any unintended clinical consequences [7,28,29].

Our PIs could finally be integrated into an existing public reporting system. In France, however, there is no public reporting of indicators at the GP practice level, but this does exist in England, with the Public Health England’s Fingertips initiative [21,30].

Very few studies have developed a set of PIs using national guidelines as the gold standard for appropriate practices, while estimating the appropriateness of antibiotic prescriptions in general practice without any information regarding clinical indication/diagnoses [3,22,23]. We are not aware of comparable studies having assessed the clinimetric properties of such a set of PIs. Some countries, however, use such PIs: for example, in Belgium, an amoxicillin/co-amoxiclav ratio is used, with an 80/20 target. In England, a trimethoprim to nitrofurantoin ratio is also used, but with only a relative reduction target [7].

We believe that other countries could adopt a similar approach and develop their own set of PIs at GP level, as our indicators seem quite easily adaptable to other settings/guidelines. Indeed, even though guidelines might vary from one country to another, the general principle underlying each PI remains the same, for example, antibiotics that are not indicated for male UTI according to national guidelines for PI 1. Countries could adapt each PI to their national context by selecting different antibiotics or setting different targets. Countries could operationalise our PIs by using their national guidelines as the standard of care and they might also select other existing QIs to be translated into PIs [3]. It is possible to compute the PI scores at region/county level to give an indication of appropriateness of prescriptions at those levels. In this way, countries that have information on prescriptions but not on prescribers could compute fractions. Such information might be more informative than the currently used overview of quantity metrics, such as defined daily doses [31]. Common indicators could be identified at European level and might be considered for inclusion in the European Surveillance of Antimicrobial Consumption Network (ESAC-Net) surveillance and benchmarking system, as is already the case for the two seasonal variation indicators we included in our work [32]. The next step should be the validation of these PIs, i.e. a comparison with QIs, based on clinical diagnoses, using a sample of antibiotic prescriptions.

A combined set of metrics, quantity metrics, PIs and Qis, is probably the best approach to assess antibiotic use, since each metric provides complementary information [3-6]. Quantity metrics and PIs are the easiest to integrate in an automated large-scale surveillance system. Coding infectious diagnoses (requiring an antibiotic or not) in electronic medical records and linking these codes to antibiotic prescriptions should be encouraged in all settings [7], since this is a prerequisite for QIs, but this is a challenge as demonstrated recently in England [33]. Periodic assessment of the accuracy of the diagnostic codes is also needed since the quality of diagnostic coding may be poor, as found in England and the United States [33,34].

Our work is original, but it has several potential limitations. First, the targets set are debatable since some are based on expert opinion; a structured consensus procedure involving a large group of stakeholders might be useful to further validate these targets [35]. Second, the NHI reimbursement database gives information on the dispensing of antibiotics. On the one hand, these data may overestimate the quantity of antibiotics really taken by the patient if compliance is suboptimal or if the package size exceeds the quantity of antibiotics prescribed, as there is no unit dispensing of antibiotics in France. On the other hand, we might underestimate prescription data if the antibiotic is not dispensed, i.e. if the patient does not go to the community pharmacy to get their treatment; a recent study conducted in north-eastern France showed that this might happen in ca 5% of antibiotic prescriptions [36]. Finally, PI 8 only reflects an estimated duration of treatment (see Supplementary Table S2 for further information), as the NHI database does not contain information on the precise prescribed daily dose or duration of treatment, but only on the dispensed packages.

In conclusion, we have defined a set of 10 proxy indicators to estimate the appropriateness of antibiotic prescriptions in general practice in north-eastern France, that are easily calculable based on reimbursement data only. These can be used within antibiotic stewardship programmes to measure and improve antibiotic prescriptions with the ultimate aim of curbing antibiotic resistance.

## Supplementary Data

811000 Supplementary Material
