# Supplementary material for: Proxy indicators to estimate appropriateness of antibiotic prescriptions by general practitioners: a proof-of-concept cross-sectional study based on reimbursement data, north-eastern France 2017
Source: Euro Surveill. 2020 Jul 9;25(27):1900468. doi: 10.2807/1560-7917.ES.2020.25.27.1900468 (PMC7364760; doi:10.2807/1560-7917.ES.2020.25.27.1900468)
Supplement: Supplementary Material [file 1900468_PULCINI_Supplement.pdf]

SUPPLEMENTARY MATERIAL (Tables S1 to S3, Figure S1)

This supplementary material is hosted by Eurosurveillance as supporting information alongside the article [Proxy indicators to estimate appropriateness of antibiotic prescriptions by general practitioners: a proof-of-concept cross-sectional study based on reimbursement data, north-eastern France 2017] on behalf of the authors who remain responsible for the accuracy and appropriateness of the content. The same standards for ethics, copyright, attributions and permissions as for the article apply. Eurosurveillance is not responsible for the maintenance of any links or email addresses provided therein.

Supplementary Table S1. Evidence base for the proxy indicators to estimate the appropriateness of systemic antibiotic prescriptions by general practitioners

Proxy indicator (PI)	Based on the following quality indicator (QI), identified in the literature review + consensus procedure[a]	French national guidelines/recommendations used to adapt the definition of the QI to the French context	French or international guidelines/recommendations used to set the target (optimal/acceptable)
**PI 1:** Antibiotic prescriptions against UTI in men (ratio)	OQI-4 Some antibiotics should be rarely prescribed	-National guidelines on Urinary Tract Infections (UTI) [1,2]<br>-Nitrofurantoin, fosfomycin-trometamol, 1st-generation quinolones (J01MB), norfloxacin, enoxacin and lomefloxacin are not recommended in male UTIs	-The optimal target is 0, but we also set an acceptable target at <0.5, based on expert opinion (since guidelines are not applicable to all patients and some of these antibiotics might be used as last-resort treatments, e.g. in complex patients with relapsing UTIs or UTIs due to multi-drug resistant bacteria)
**PI 2:** Antibiotic prescriptions against UTI in women (ratio)	OQI-3 Outpatients should receive antibiotic therapy compliant with guidelines	-National guidelines on Urinary Tract Infections (UTIs) [1,2]	-Cystitis are much more frequent in primary care as compared to pyelonephritis. Regarding cystitis in women, fluoroquinolones are never first-line treatments (whereas this might be the case for empirical treatment for pyelonephritis). In female cystitis, nitrofurantoin, pivmecillinam and fosfomycin-trometamol are the usual 1st-line treatments (depending on the clinical situation). Moreover, nitrofurantoin, pivmecillinam and fosfomycin-trometamol are exclusively recommended for UTIs. This is not the case for fluoroquinolones, which can be indicated in very selected indications (e.g. levofloxacin is recommended as second-line treatment in some respiratory infections), but the level of use of fluoroquinolones should be very limited if guidelines are complied with. [3]<br>-For all these reasons, we selected a ratio >1, based on expert opinion, even though the optimal ratio might be even higher
**PI 3:** Repeated prescription of quinolones (%)	OQI-3 Outpatients should receive antibiotic therapy compliant with guidelines	-National guidelines on Urinary Tract Infections (UTI) and lower respiratory tract infections [1,2]<br>-National recommendations on fluoroquinolone use [3]<br>-Based on these recommendations, a quinolone should not be used (whenever possible) among patients having been prescribed a quinolone in the preceding 6 months	-The optimal target is 0, but we also set an acceptable target at <10%, based on expert opinion (since guidelines are not applicable in all cases)
**PI 4:** Seasonal variation of total antibiotic prescriptions (%)	OQI-2 Antibiotics should not be prescribed for viral infections or (most) self-limiting bacterial infections	-Published articles [4-6]<br>-ESAC-Net [7]	We set a target <20% based on expert opinion and the range of existing European data. [7]

**PI 5:** Seasonal variation of quinolone prescriptions (%)	OQI-2 Antibiotics should not be prescribed for viral infections or (most) self-limiting bacterial infections	-Published articles [4-6]<br>-ESAC-Net [7]	A target ≤5% is used in Scotland as a national indicator. [8]<br>We also set an acceptable target at <10%, based on expert opinion and the range of existing European data. [7]
**PI 6:** Amoxicillin / second-line antibiotics prescriptions (ratio)	OQI-3 Outpatients should receive antibiotic therapy compliant with guidelines	-National guidelines on the most common infections encountered in primary care [1,2]	-In France, 70% of all antibiotics prescribed in primary care concern upper and lower respiratory tract infections. [9]<br>-Amoxicillin is the 1st-line recommended treatment for almost all bacterial respiratory tract infections, whereas co-amoxiclav, cephalosporins and macrolides are usually 2nd-line treatments<br>-For all these reasons, we selected a ratio >1, based on expert opinion, even though the optimal ratio might be even higher
**PI 7:** Prescription of not indicated antibiotics (%)	OQI-4 Some antibiotics should be rarely prescribed	-National guidelines on the most common infections encountered in primary care [1,2]<br>- Lomefloxacin, moxifloxacin, 1st-generation quinolones (J01MB), norfloxacin, enoxacin and lomefloxacin, telithromycin, spiramycin-metronidazole and cefaclor are not indicated according to these national guidelines	-The optimal target is 0, but we also set an acceptable target at <0.5, based on expert opinion (since guidelines are not applicable to all patients and some of these antibiotics might be used as last-resort treatments)
**PI 8:** Estimated duration of antibiotic prescriptions > 8 days (%)	OQI-3 Outpatients should receive antibiotic therapy compliant with guidelines	-National guidelines on the most common infections encountered in primary care [1,2]<br>-The maximum recommended duration of antibiotic treatment for almost all bacterial infections encountered in GP practice is one week, and durations <1 week are common<br>-We selected here antibiotics that are almost exclusively recommended in respiratory tract and skin infections, or cystitis, where recommended durations <8 days are the rule	-We set an optimal target at <5% for durations of 9 days or more, and an acceptable target at <10%, based on expert opinion<br>-In France, unit dispensing is not in place, so antibiotics are delivered using packages. The packages' size is however adapted to the most frequent durations advised in national recommendations<br>- See supplementary Table 2 for more details regarding the calculation of this indicator
**PI 9:** Co-prescription of antibiotic and non-steroidal anti-inflammatory drugs (NSAIDs) (%)	OQI-3 Outpatients should receive antibiotic therapy compliant with guidelines	-NSAIDs are never indicated and should be avoided in bacterial infections encountered in primary care, except for fever in children not controlled by paracetamol. [10]<br>-National guidelines on the most common infections encountered in primary care [1,2]<br>-This indicator does not take into account self-medication with NSAIDs (some NSAIDs can be bought at the patient's cost in community pharmacies without a prescription, and are therefore not recorded in the NHI database)	-The optimal target is 0, but we also set an acceptable target at <5%, based on expert opinion (since guidelines are not applicable to all patients, and some patients might be on NSAIDs for another reason)

**PI 10:** Co-prescription of antibiotic and corticosteroids (%)	OQI-3 Outpatients should receive antibiotic therapy compliant with guidelines	-Corticosteroids are never indicated and should be avoided in bacterial infections encountered in primary care, except for blocked acute bacterial sinusitis (which is very rare and usually managed by ENT specialists)<br>-National guidelines on the most common infections encountered in primary care [1,2]	-The optimal target is 0, but we also set an acceptable target at <5%, based on expert opinion (since guidelines are not applicable to all patients, and some patients might be on corticosteroids for another reason)

[a] J Antimicrob Chemother. 2018;73(suppl_6):vi40-vi49

Supplementary Table S2. Detailed procedure for the calculation of Proxy Indicator n°8: Estimated duration of antibiotic prescriptions > 8 days

In France, unit dispensing is not in place, so antibiotics are delivered using packages. The packages' sizes available on the French market should however be adapted by pharmaceutical companies to the most frequent durations advised in national recommendations; an experiment conducted in 100 community pharmacies in France showed that per-unit dispensing reduced by 10% the number of pills supplied.[11] To account for this excess of dispensed pills as compared to the exact duration prescribed by the GP, we set a target at 9 days or more of treatment, instead of 8 days or more (see Supplementary Table S1 for the rationale for 8 days).

The French National Health Insurance database does not contain information on the precise prescribed daily dose or duration, but only on the dispensed packages. We here calculated the quantity of dispensed antibiotics for each prescription (number of packages multiplied by the quantity of antibiotic [in grams] per package).

We then determined a 'usually recommended total daily dose' for the most frequently prescribed antibiotics (see details in the Table below). This usual total daily dose is the most commonly prescribed daily dose in general practice in adults, according to national guidelines. [1,2] We did not plan initially to restrict this metric to adults > 16 years old, since: (i) GPs usually take care of few children; (ii) the total daily dose recommended in children > 40-50 kg is usually the same as for adults; and (iii) we planned to explore differences according to age classes in our case-mix analyses.

We excluded the following antibiotics: (i) azithromycin and fosfomycin-trometamol, since both are recommended for short durations given their long half-life; and (ii) doxycycline and fluoroquinolones since these antibiotics might be quite frequently prescribed for durations of more than one week according to national recommendations.

We acknowledge that this indicator only approximates the real duration prescribed by the general practitioner (GP), but data on days-of-therapy are not available in routine in existing French databases. We might therefore:

- overestimate the real duration prescribed by the GP if the quantity dispensed exceeds the quantity prescribed by the GP (in case the packages do not match the exact quantity prescribed) or if the total daily dose prescribed by the GP exceeds the usual prescribed total daily dose considered here (see Table below)
- underestimate the real duration if the total daily dose prescribed by the GP is lower than the usual prescribed total daily dose considered here (see Table below)

Antibiotic	Total cumulated 'usually recommended total daily dose' corresponding to a duration of treatment > 8 days
Amoxicillin (J01CA04)	> 24 grams
Co-amoxiclav (J01CR02)	> 24 grams
Cefuroxime (J01DC02)<br>Cefpodoxime (J01DD13)	> 4 grams<br>> 3.2 grams
Roxithromycin (J01FA06)<br>Clarithromycin (J01FA09)	> 2.4 grams<br>> 8 grams
Pristinamycin (J01FG01)	> 24 grams
Nitrofurantoin (J01FG01)	> 2.4 grams

Supplementary Table S3. Results for the ten proxy indicators to estimate the appropriateness of systemic antibiotic prescriptions, calculated at the general practitioner level, compared to existing published data

Proxy indicator (PI) in our study	Target value	Mean ± SD	Median (IQR)	% of GPs who reached the target (performance)	Comparison to literature/existing published data (including reference n°12)
**PI 1:** Antibiotic prescriptions against UTI in men (ratio)	Optimal: 0<br>Acceptable: <0.5	0.5 ± 0.8	0.2 (0 ; 0.5)	Optimal: 46.6%<br>Acceptable: 73.0%	**Reference n°13**<br>Exactly the same indicator, but calculated in adult patients of GPs in 2009 in South-Eastern France, and in Defined Daily Doses=DDDs (not prescriptions)<br>Mean 17.99 ± 9.40<br>Median 16.77 (IQR 11.97; 22.30)<br>Optimal : 1.2%
**PI 2:** Antibiotic prescriptions against UTI in women (ratio)	>1	3.4 ± 5.0	2.1 (1.0 ; 4.0)	75.0%	**Reference n°14**<br>Bourgogne and Franche-Comté regions in 2016, 15 to 65 yo women, DDDs<br>Not exactly the same indicator: (fosfomycin + pivmecillinam + nitrofurantoin) / (norfloxacin + ciprofloxacin + ofloxacin)<br>Ranged from 0.29 to 0.96 depending on the department
**PI 3:** Repeated prescription of quinolones (%)	Optimal: 0<br>Acceptable: <10%	16.8 ± 11.2	16.7 (8.7 ; 24.1)	Optimal: 13.6%<br>Acceptable: 27.6%	**Reference n°13**<br>Not exactly the same indicator; calculated in adult patients of GPs in 2009 in South-Eastern France, in DDDs<br>Prescriptions of levofloxacin, ofloxacin or ciprofloxacin (J01MA12+01+02) among patients having been prescribed a quinolone (J01M) in the preceding 6 months<br>Mean 9.79 ± 8.63<br>Median 8.41 (IQR 3.70; 14.12)<br>Optimal: 20.3%
**PI 4:** Seasonal variation of total antibiotic prescriptions (%)	<20%	85.8 ± 411.7	52.4 (35.9 ; 69.6)	10.7%	**Reference n°15**<br>Calculated in adult patients of GPs in 2009 in South-Eastern France, in DDDs<br>Median 49.2%;<br><br>**Reference n°16**<br>Calculated in <16 yo children patients of GPs in 2009 in South-Eastern France, in number of prescriptions<br>Median 66.7%<br><br>**Reference n°7**<br>Ranges from 11.4% to 41.1% depending on the European country (no data available for France); DDDs
**PI 5:** Seasonal variation of	Optimal: < 5%<br>Acceptable: <10%			Optimal: 34.8%<br>Acceptable: 38.2%	**Reference n°15**<br>Calculated in adult patients of GPs in 2009 in South-Eastern France, in DDDs

quinolone prescriptions (%)		49.9 ± 138.3	25.0 (-6.7 ; 71.4)		Median 25.0%<br><br>**Reference n°7**<br>Ranges from 2.5% to 32.3% depending on the European country (no data available for France); DDDs
**PI 6:**<br>Amoxicillin / second-line antibiotics prescriptions (ratio)	>1	0.9 ± 0.7	0.8 (0.5 ; 1.2)	35.6%	**Reference n°14**<br><15 yo children, DDDs<br>Different ratio: amoxicillin / (co-amoxiclav + cefixime + cefpodoxime)<br>Ranges between 1.1 and 2.0 depending on the department<br><br>**Reference n°17**<br>Not exactly the same metric: percentage of broad-spectrum prescribed antibiotic items (cephalosporin, quinolone and co-amoxiclav class); prescribed items<br>England Q1 2018 = 8.1%
**PI 7:**<br>Prescription of not indicated antibiotics (%)	Optimal target: 0<br>Acceptable target: <0.5%	3.3 ± 3.7	2.3 (0.9 ; 4.3)	Optimal: 7.2%<br>Acceptable: 14.6%	**Reference n°13**<br>Not the same indicator; calculated in adult patients of GPs in 2009 in South-Eastern France, in DDDs<br>Prescriptions of first-generation quinolones (J01MB), expressed as a percentage of the total prescriptions of antibacterials for systemic use (J01)<br>Mean 0.32 ± 1.21<br>Median 0 (0; 0)<br>Optimal: 75.2%
**PI 8:**<br>Estimated duration of antibiotic prescriptions > 8 days (%)	Optimal: <5%<br>Acceptable: <10%	19.6 ± 11.1	18.0 (11.1 ; 26.3)	Optimal: 2.4%<br>Acceptable: 9.1%	No comparable data found
**PI 9:**<br>Co-prescription of antibiotic and non-steroidal anti-inflammatory drugs (NSAIDs) (%)	Optimal: 0<br>Acceptable: <5%	13.3 ± 10.1	10.8 (5.8 ; 18.6)	Optimal: 1.4%<br>Acceptable: 20.9%	No comparable data found
**PI 10:**<br>Co-prescription of antibiotic and corticosteroids (%)	Optimal: 0<br>Acceptable target: <5%	16.1 ± 11.6	13.4 (7.6 ; 21.8)	Optimal: 1.7%<br>Acceptable: 13.4%	No comparable data found

Abbreviations. SD = standard deviation; IQR = interquartile range; GP = general practitioner

REFERENCES

1. http://www.infectiologie.com/fr/recommandations.html

2. https://antibioclic.com

3. http://www.infectiologie.com/UserFiles/File/medias/Recos/2015-MAP-fluoroquinolones-SPILF.pdf

4. Versporten A, Gyssens IC, Pulcini C, et al. Metrics to assess the quantity of antibiotic use in the outpatient setting: a systematic review followed by an international multidisciplinary consensus procedure. J Antimicrob Chemother 2018; 73(suppl_6): vi59-vi66.

5. Le Maréchal M, Tebano G, Monnier AA, et al. Quality indicators assessing antibiotic use in the outpatient setting: a systematic review followed by an international multidisciplinary consensus procedure. J Antimicrob Chemother 2018; 73(suppl_6): vi40-vi49.

6. Coenen S, Ferech M, Haaijer-Ruskamp FM, et al. European Surveillance of Antimicrobial Consumption (ESAC): quality indicators for outpatient antibiotic use in Europe. Qual Saf Health Care. 2007; 16: 440-5.

7. https://ecdc.europa.eu/en/antimicrobial-consumption/database/quality-indicators

8. Nathwani D, Sneddon J, Patton A, Malcolm W. Antimicrobial stewardship in Scotland: impact of a national programme. Antimicrob Resist Infect Control. 2012; 1: 7.

9. https://ansm.sante.fr/S-informer/Points-d-information-Points-d-information/Evolution-des-consommations-d-antibiotiques-en-France-entre-2000-et-2015-Point-d-Information

10. https://www.has-sante.fr/portail/jcms/c_2674284/fr/prise-en-charge-de-la-fievre-chez-l-enfant

11. Treibich C, Lescher S, Sagaon-Teyssier L, Ventelou B. The expected and unexpected benefits of dispensing the exact number of pills. PLoS One. 2017; 12(9): e0184420.

12. Zanichelli V, Monnier AA, Gyssens IC, et al. Variation in antibiotic use among and within different settings: a systematic review. J Antimicrob Chemother. 2018 Jun 1;73(suppl_6):vi17-vi29.

13. Pulcini C, Lions C, Ventelou B, Verger P. Approaching the quality of antibiotic prescriptions in primary care using reimbursement data. Eur J Clin Microbiol Infect Dis 2013; 32: 325-32.

14. https://projet.chu-besancon.fr/rfclin/surveillance/observatoire/oscar_rapport_ville_2016.pdf

15. Pulcini C, Lions C, Ventelou B, Verger P. Drug-specific quality indicators assessing outpatient antibiotic use among French general practitioners. Eur J Public Health. 2013; 23: 262-4.

16. Pulcini C, Lions C, Ventelou B, Verger P. Indicators show differences in antibiotic use between general practitioners and paediatricians. Eur J Clin Microbiol Infect Dis 2013; 32: 929-35.

17. https://fingertips.phe.org.uk/profile/amr-local-indicators/

Supplementary Figure S1. Distribution of the ten proxy indicators values among GPs

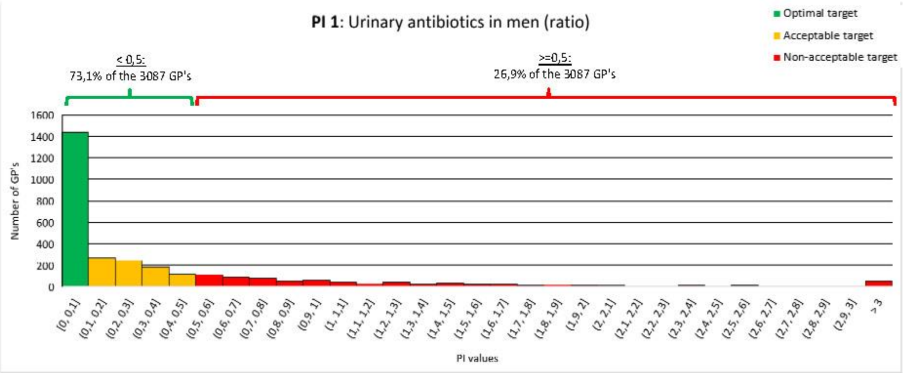

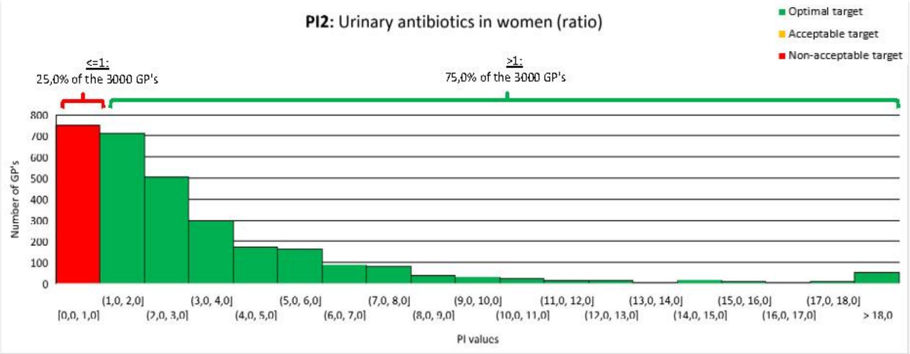

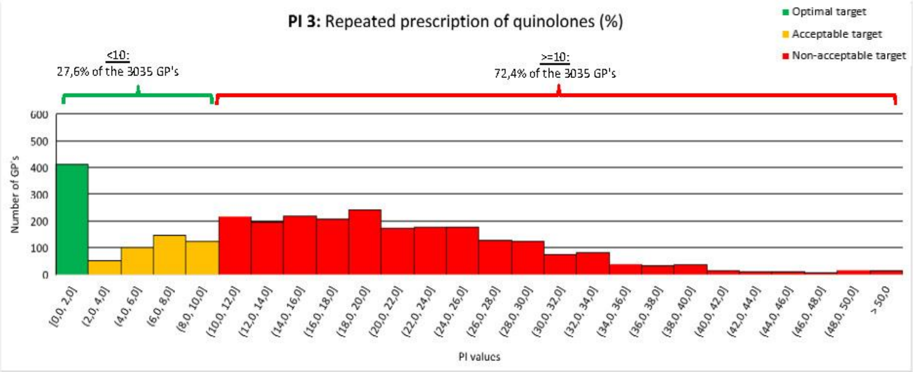

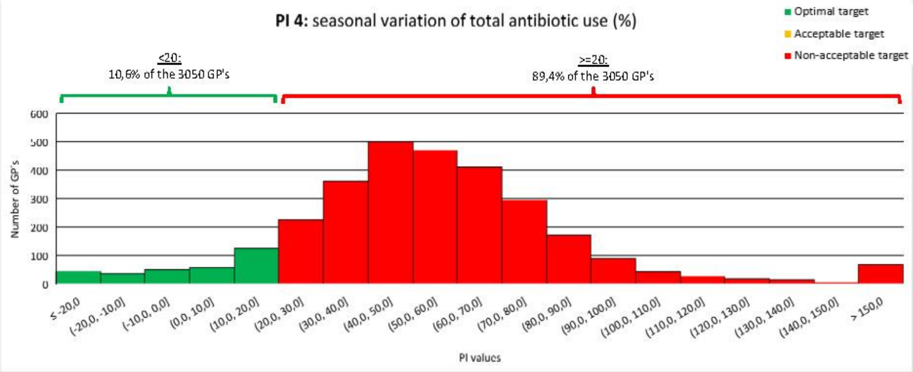

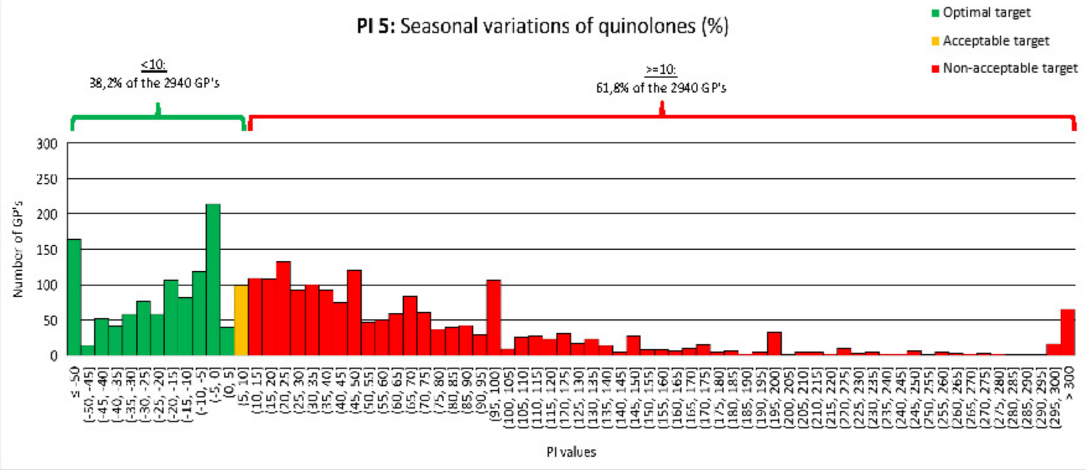
PI 5: Seasonal variations of quinolones (%)
Optimal target
Acceptable target
Non-acceptable target
<10:
38,2% of the 2940 GP's
>=10:
61,8% of the 2940 GP's
Number of GP's
PI values

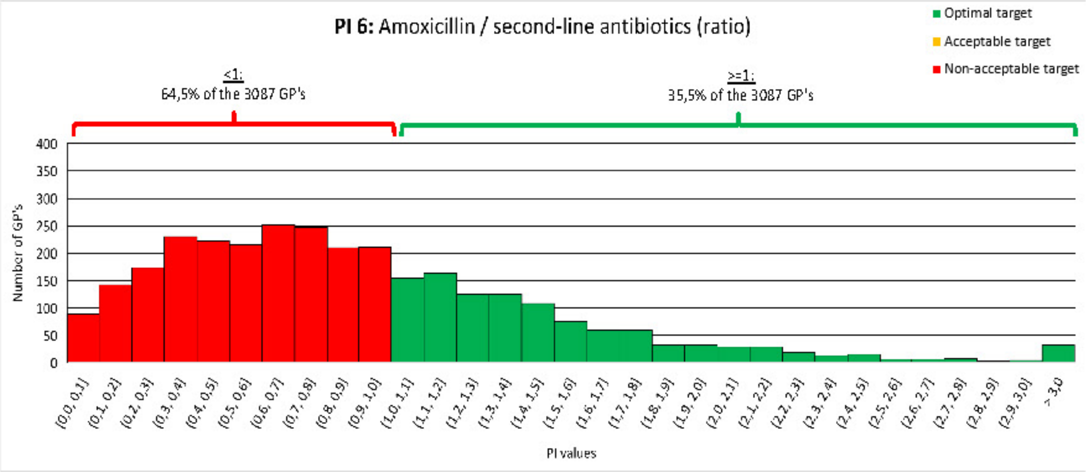
PI 6: Amoxicillin / second-line antibiotics (ratio)
Optimal target
Acceptable target
Non-acceptable target
<1:
64,5% of the 3087 GP's
>=1:
35,5% of the 3087 GP's
Number of GP's
PI values

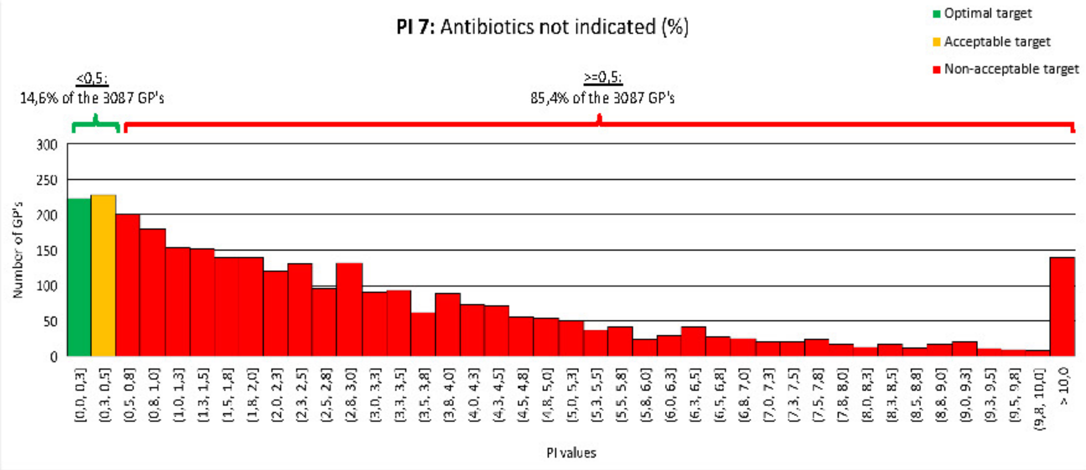
PI 7: Antibiotics not indicated (%)
Optimal target
Acceptable target
Non-acceptable target
<0,5:
14,6% of the 3087 GP's
>=0,5:
85,4% of the 3087 GP's
Number of GP's
PI values

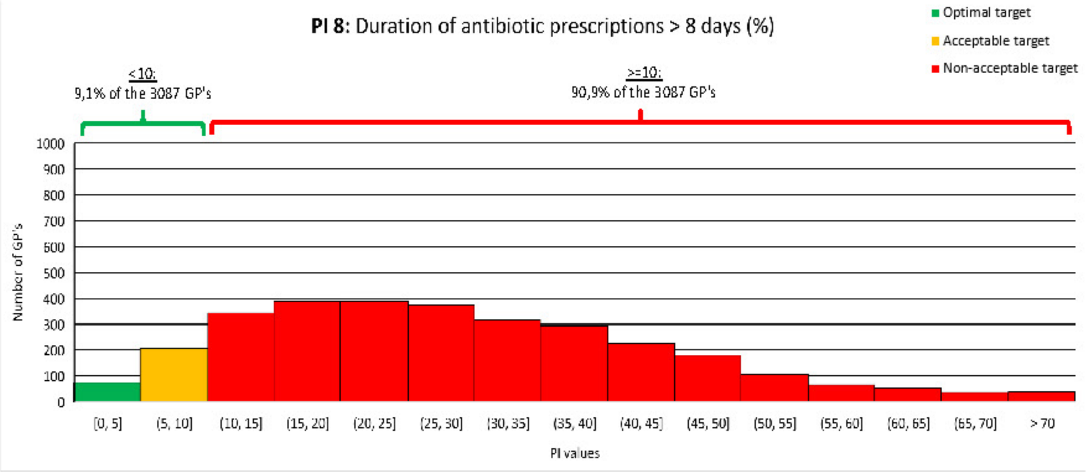

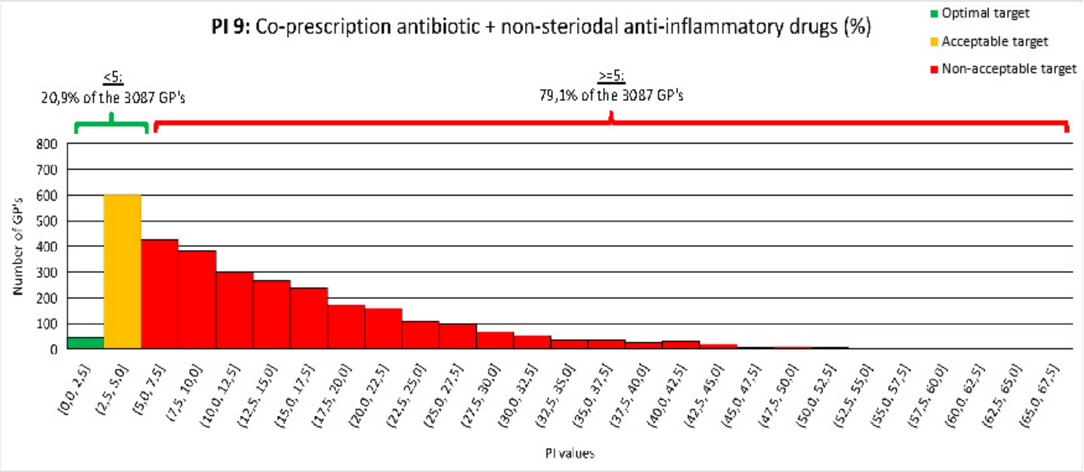

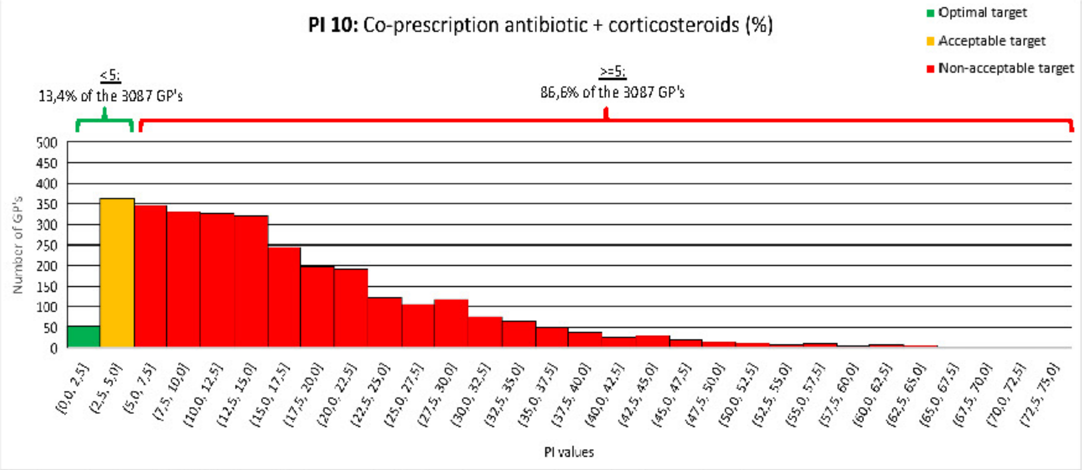

Supplementary figure 1. Distribution of the ten proxy indicators values among the sample of general practioners
